# Supplementary material for: SARS-CoV-2 Omicron variant causes mild pathology in the upper and lower respiratory tract of hamsters
Source: Nat Commun. 2022 Jun 20;13:3519. doi: 10.1038/s41467-022-31200-y (PMC9207884; doi:10.1038/s41467-022-31200-y)
Supplement: Supplementary file 1 — Supplementary Information [file 41467_2022_31200_MOESM1_ESM.docx]

**Supplementary information**

**SARS-CoV-2 Omicron variant causes mild pathology in the upper and lower respiratory tract of hamsters**

Federico Armando^1,*^, Georg Beythien^1,*^, Franziska K. Kaiser^2,*^, Lisa Allnoch^1^, Laura Heydemann^1^, Malgorzata Rosiak^1^, Svenja Becker^1^, Mariana Gonzalez-Hernandez^2^, Mart M. Lamers^3^, Bart L. Haagmans^3^, Kate Guilfoyle^4^, Geert van Amerongen^4^, Malgorzata Ciurkiewicz^1,#^, Albert D.M.E. Osterhaus^2,5,#^, Wolfgang Baumgärtner^1,#^

^1^Department of Pathology, University of Veterinary Medicine, Foundation, Hanover, Germany

^2^Research Center for Emerging Infections and Zoonoses, University of Veterinary Medicine, Hanover, Germany

^3^Department of Viroscience, Erasmus MC, Rotterdam, Netherlands

^4^Viroclinics Xplore, Schaijk, Netherlands.

^5^Global Virus Network, Center of Excellence

^*, # authors contributed equally as first and last authors^

Corresponding author:

Prof. Dr. W. Baumgärtner, Ph.D. ([Wolfgang.Baumgaertner@tiho-hannover.de](mailto:Wolfgang.Baumgaertner@tiho-hannover.de))

Department of Pathology,

University of Veterinary Medicine, Foundation, Hanover

Bünteweg 17,

Hannover 30559, Germany.

Email: Wolfgang.Baumgaertner@tiho-hannover.de

This file contains:

Supplementary figures: 3

Supplementary tables: 5

**
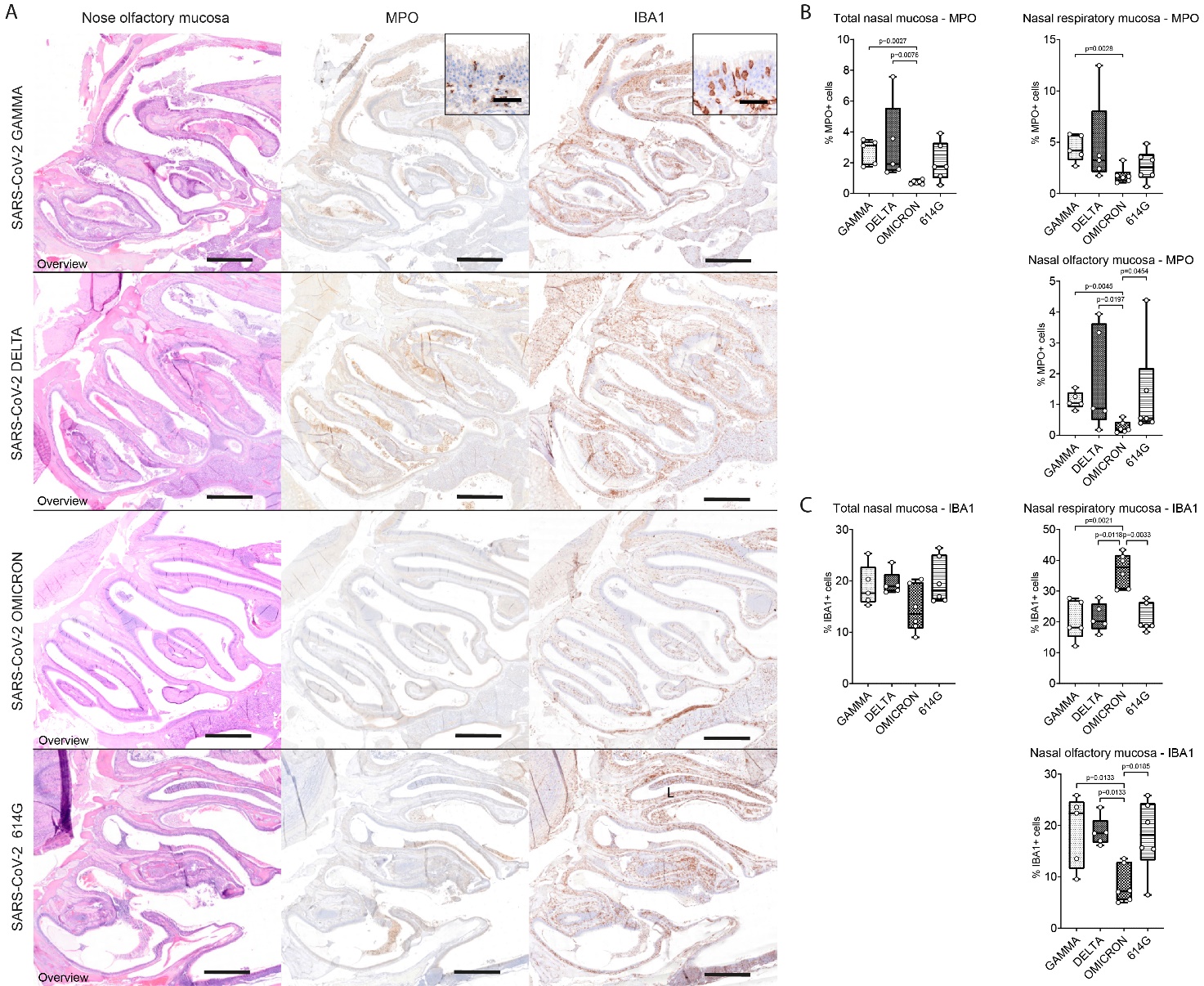
**

**Supplementary Figure 1. Decreased inflammatory cell infiltration in the nasal turbinates of VOC Omicron infected hamsters.**

A) Representative images showing an overview of the caudal nasal turbinates of hamsters infected with VOCs Gamma, Delta, Omicron or 614G strain. The left panel shows hematoxylin and eosin (HE) stained sections. The middle and right panels show immunolabeling for myeloperoxidase (MPO, marker for neutrophils/heterophils) and Ionized calcium-binding adapter molecule 1 (IBA1, marker for macrophages/histiocytic cells), respectively. The inserts in the top panel show a detailed view of intraepithelial, immunolabeled cells (brown signal). VOC Omicron infected animals show lower numbers of immunolabeled cells in the caudal nasal turbinates, which are mostly covered by olfactory mucosa. Scale bars: 1 mm (overviews) and 20 µm (insets). B) Quantification of MPO^+^ in the total nasal mucosa as well as the respiratory and olfactory compartment shows lowest numbers of immunolabeled cells in VOC Omicron infected hamsters. C) Quantification of IBA1^+^ in the total nasal mucosa as well and in the olfactory compartment shows lowest numbers of macrophages/histiocytic cells in VOC Omicron infected hamsters. B, C) Data are shown as box and whisker plots. The bounds of the box plot indicate the 25th and 75th percentiles, the bar indicates medians, and the whiskers indicate minima and maxima. Data was tested by two-tailed Mann-Whitney-U tests followed by Benjamini-Hochberg correction. A p-value of ≤ 0.05 was chosen as the cut-off for statistical significance. N= 5 animals/group for VOCs Gamma and Delta and 6 animals/group for VOC Omicron and 614G. For quantification, one entire longitudinal section of the nasal turbinates was evaluated per animal. Cells were quantified exclusively within the mucosa, intraluminal debris was excluded from analysis. Source data are provided as a Source Data file.


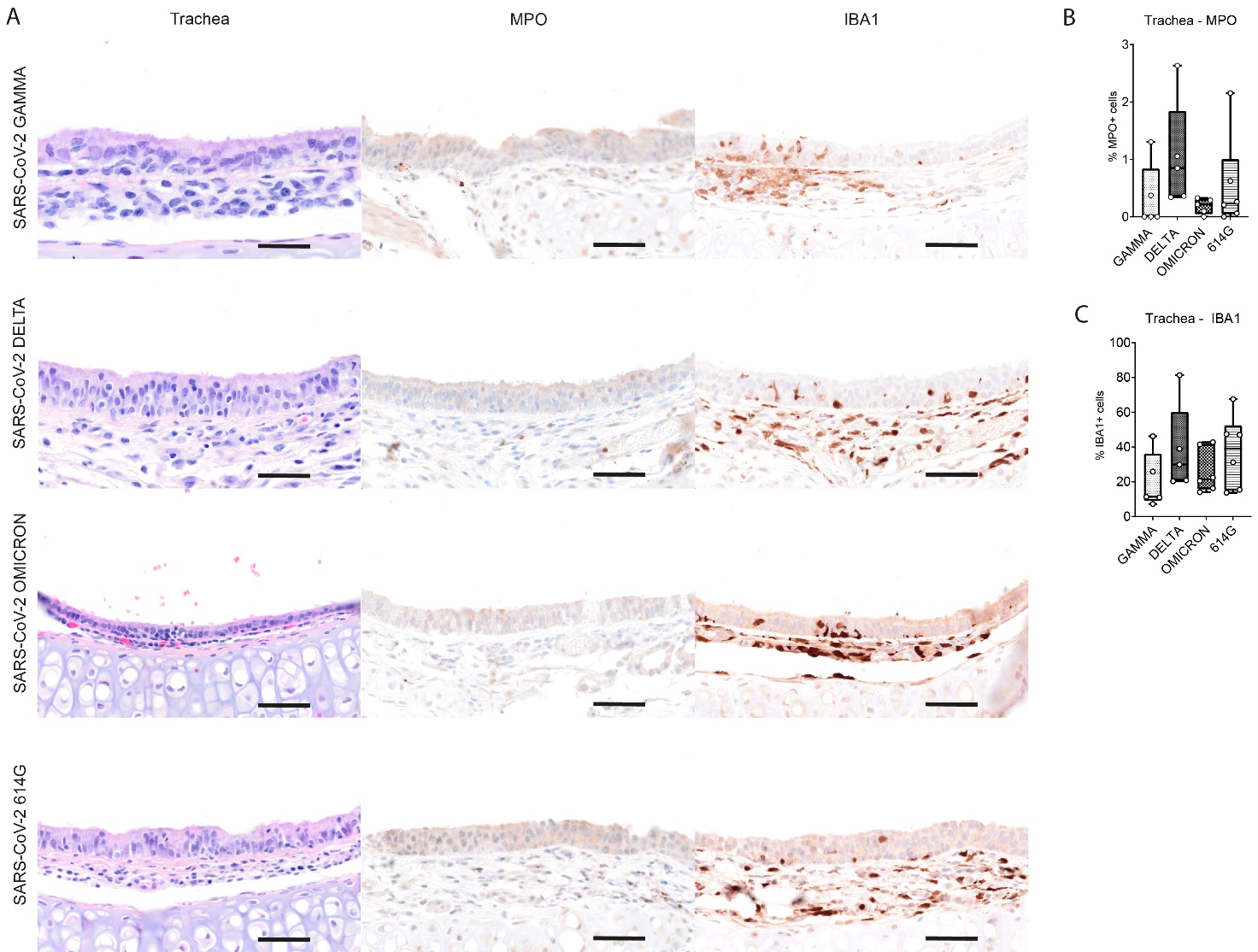


**Supplementary Figure 2. Inflammatory cell infiltration in the trachea is comparable in hamsters infected with different strains of SARS-CoV-2.**

A) Representative images showing tracheal mucosa of hamsters infected with VOCs Gamma, Delta, Omicron or 614G strain. The left panel shows hematoxylin and eosin (HE) stained sections. The middle and right panels show immunolabeling for myeloperoxidase (MPO, marker for neutrophils/heterophils) and Ionized calcium-binding adapter molecule 1 (IBA1, marker for macrophages/histiocytic cells), respectively. Hamsters infected with different SARS-CoV-2 strains show low numbers of MPO^+^ neutrophils/heterophils and moderate numbers of IBA1+ macrophages/histiocytes in the epithelium and the lamina propria (brown signal). Scale bars: 50 µm. B-C) Quantification of MPO (B) and IBA1 (C) shows no significant differences among the infection groups. Data are shown as box and whisker plots. The bounds of the box plot indicate the 25th and 75th percentiles, the bar indicates medians, and the whiskers indicate minima and maxima. Data was tested by two-tailed Mann-Whitney-U tests followed by Benjamini-Hochberg correction. A p-value of ≤ 0.05 was chosen as the cut-off for statistical significance. N = 5 animals/group for VOCs Gamma and Delta and 6 animals/group for VOC Omicron and 614G. For quantifications, one entire cross- and one entire longitudinal section were evaluated per animal. Source data are provided as a Source Data file.

**
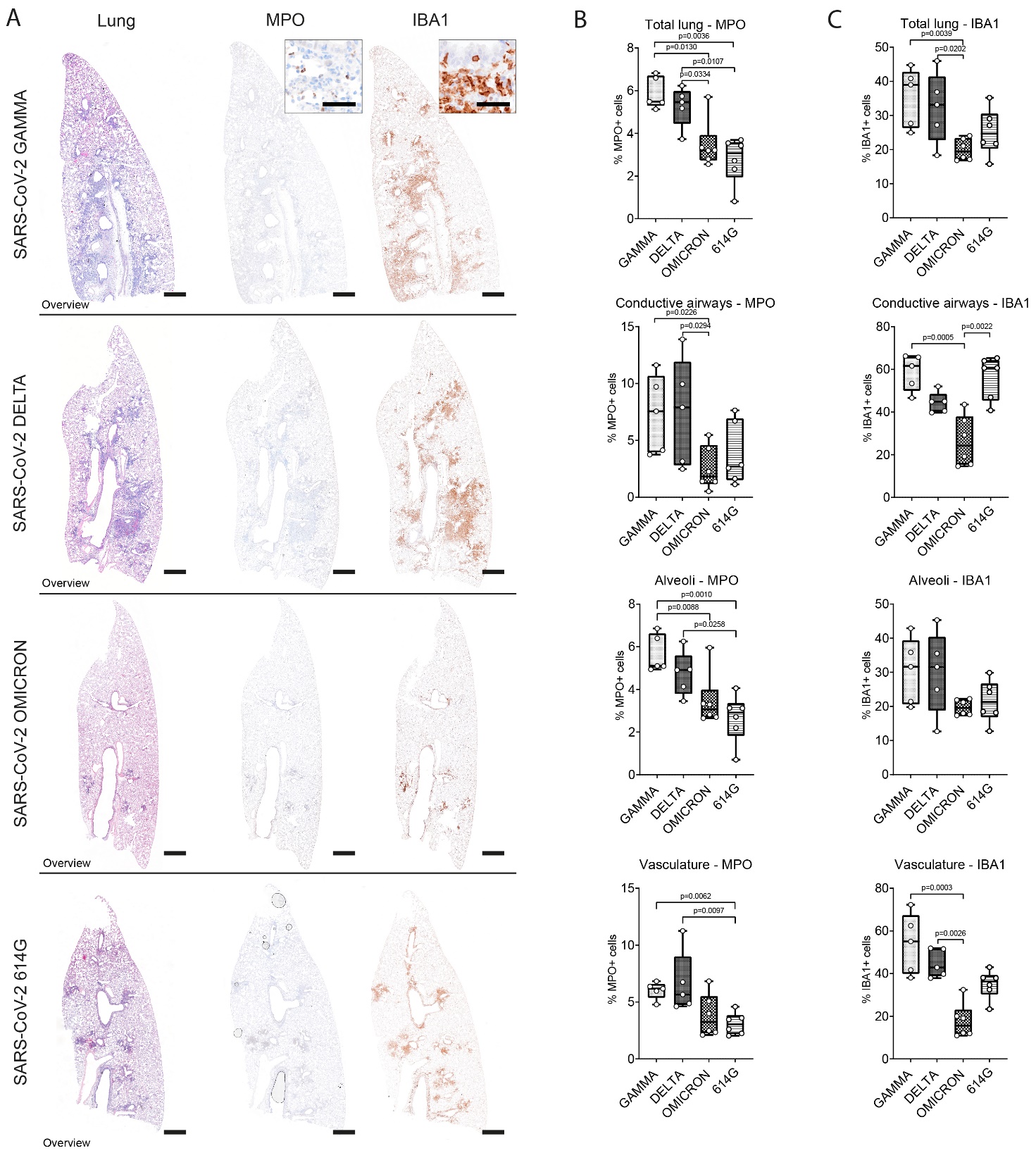
**

**Supplementary Figure 3. VOC Omicron infected hamsters show decreased inflammatory cell infiltrates in the lung compared to hamsters infected with other SARS-CoV-2 strains.**

A) Representative images showing the overview of left lung lobes of hamsters infected with VOCs Gamma, Delta, Omicron or 614G strain. The left panel shows hematoxylin and eosin (HE) stained sections. The middle and right panels show immunolabelings for myeloperoxidase (MPO, marker for neutrophils/heterophils) and Ionized calcium-binding adapter molecule 1 (IBA1, marker for macrophages/histiocytic cells), respectively. The inserts in the top panel show a detailed view immunolabeled cells (brown signal). Scale bars: 1 mm (overview) and 20 µm (insets). B) Quantification of MPO in different lung compartments shows lower numbers of neutrophils/heterophils in hamsters infected with VOC Omicron or the 614G strain compared to hamsters infected with VOCs Gamma and Delta. C) Quantification of IBA1 shows lowest numbers of macrophages/histiocytic cells in the total lung, conductive airways and vessels of VOC Omicron infected hamsters compared to other groups. B,C) Data are shown as box and whisker plots. The bounds of the box plot indicate the 25th and 75th percentiles, the bar indicates medians, and the whiskers indicate minima and maxima. Data was tested by two-tailed Mann-Whitney-U tests followed by Benjamini-Hochberg correction. A p-value of ≤ 0.05 was chosen as the cut-off for statistical significance. N = 5 animals/group for VOCs Gamma and Delta and 6 animals/group for VOC Omicron and 614G. For quantifications, one entire longitudinal section of the left lung lobe was evaluated. Source data are provided as a Source Data file.

**Supplementary Table 1: Result of statistical analysis of body weight**: Group medians and exact p-values ≤0,05 obtained by two-tailed Mann-Whitney-U tests, followed by Benjamini-Hochberg-correction for multiple testing are given.

|  | **Group** | **Median** | SARS-CoV-2 Gamma | SARS-CoV-2 Delta | SARS-CoV-2 Omicron | SARS-CoV-2 614G |
| --- | --- | --- | --- | --- | --- | --- |
| **Body weight [%]** | |  |  |  |  |  |
|  | SARS-CoV-2 Gamma | 94.28 | **-** | ns | 0.0053 | ns |
|  | SARS-CoV-2 Delta | 93.98 | ns | **-** | 0.0038 | ns |
|  | SARS-CoV-2 Omicron | 100.8 | 0.0053 | 0.0038 | **-** | ns |
|  | SARS-CoV-2 614G | 97.57 | ns | ns | ns | **-** |

ns = not significant

**Supplementary Table 2: Result of statistical analysis of lesions and viral load in the nasal turbinates**: Group medians and exact p-values ≤0.05 obtained by two-tailed Mann-Whitney-U tests, followed by Benjamini-Hochberg-correction for multiple testing, are given.

|  | **Group** | **Median** | SARS-CoV-2 Gamma | SARS-CoV-2 Delta | SARS-CoV-2 Omicron | SARS-CoV-2 614G |
| --- | --- | --- | --- | --- | --- | --- |
| **Total nasal mucosa lesions** | |  |  |  |  |  |
|  | SARS-CoV-2 Gamma | 14 | **-** | ns | 0.0085 | ns |
|  | SARS-CoV-2 Delta | 13 | ns | **-** | 0.0210 | ns |
|  | SARS-CoV-2 Omicron | 9 | 0.0085 | 0.0210 | **-** | 0.0015 |
|  | SARS-CoV-2 614G | 15 | ns | ns | 0.0015 | **-** |
| **Nasal respiratory mucosa lesions** | |  |  |  |  |  |
|  | SARS-CoV-2 Gamma | 7 | **-** | ns | 0.0054 | ns |
|  | SARS-CoV-2 Delta | 7 | ns | **-** | 0.0429 | ns |
|  | SARS-CoV-2 Omicron | 6 | 0.0054 | 0.0429 | **-** | 0.0041 |
|  | SARS-CoV-2 614G | 8 | ns | ns | 0.0041 | **-** |
| **Nasal olfactory mucosa lesions** | |  |  |  |  |  |
|  | SARS-CoV-2 Gamma | 4 | **-** | ns | 0.0301 | ns |
|  | SARS-CoV-2 Delta | 4 | ns | **-** | 0.0301 | ns |
|  | SARS-CoV-2 Omicron | 0.5 | 0.0301 | 0.0301 | **-** | ≤0.0001 |
|  | SARS-CoV-2 614G CoV-2 614G | 8 | ns | ns | ≤0.0001 | **-** |
| **Nasal respiratory mucosa - SARS-CoV-2 NP [% cells]** | | | |  |  |  |
|  | SARS-CoV-2 Gamma | 1.024 | **-** | ns | ns | ns |
|  | SARS-CoV-2 Delta | 0.7954 | ns | **-** | ns | ns |
|  | SARS-CoV-2 Omicron | 1.641 | ns | ns | **-** | ns |
|  | SARS-CoV-2 614G | 1.349 | ns | ns | ns | **-** |
| **Nasal olfactory mucosa - SARS-CoV-2 NP [% cells]** | | |  |  |  |  |
|  | SARS-CoV-2 Gamma | 5.567 | **-** | ns | ns | ns |
|  | SARS-CoV-2 Delta | 4.241 | ns | **-** | ns | 0.0300 |
|  | SARS-CoV-2 Omicron | 2.226 | ns | ns | **-** | 0.0006 |
|  | SARS-CoV-2 614G | 17.94 | ns | 0.0300 | 0.0006 | **-** |
| **Nose - TCID50 [TCID50/ml]** | |  |  |  |  |  |
|  | SARS-CoV-2 Gamma | 1780000 | **-** | 0.0218 | ≤0.0001 | ns |
|  | SARS-CoV-2 Delta | 31600 | 0.0218 | **-** | ns | ns |
|  | SARS-CoV-2 Omicron | 2470 | ≤0.0001 | ns | **-** | 0.0024 |
|  | SARS-CoV-2 614G | 492000 | ns | ns | 0.0024 | **-** |

**Supplementary Table 2 (continued): Result of statistical analysis of lesions and viral load in the nasal turbinates**: Group medians and exact p-values ≤0.05 obtained by two-tailed Mann-Whitney-U tests, followed by Benjamini-Hochberg-correction for multiple testing, are given.

| **Group** | | **Median** | SARS-CoV-2 Gamma | SARS-CoV-2 Delta | SARS-CoV-2 Omicron | SARS-CoV-2 614G |
| --- | --- | --- | --- | --- | --- | --- |
| **Nose – SARS-CoV-2 RNA [Ct]** | |  |  |  |  |  |
|  | SARS-CoV-2 Gamma | 12.07 | **-** | ns | ≤0.0001 | 0.0124 |
|  | SARS-CoV-2 Delta | 15.07 | ns | **-** | 0.0113 | ns |
|  | SARS-CoV-2 Omicron | 23.34 | ≤0.0001 | 0.0113 | **-** | ns |
|  | SARS-CoV-2 614G | 16.81 | 0.0124 | ns | ns | **-** |

| **Total nasal mucosa – IBA1 [% cells]** | | | |  |  |  |
| --- | --- | --- | --- | --- | --- | --- |
|  | SARS-CoV-2 Gamma | 18.960 | **-** | ns | ns | ns |
|  | SARS-CoV-2 Delta | 19.530 | ns | **-** | ns | ns |
|  | SARS-CoV-2 Omicron | 14.530 | ns | ns | **-** | ns |
|  | SARS-CoV-2 614G | 19.990 | ns | ns | ns | **-** |
| **Nasal respiratory mucosa – IBA1 [% cells]** | | |  |  |  |  |
|  | SARS-CoV-2 Gamma | 20.450 | **-** | ns | 0.0021 | ns |
|  | SARS-CoV-2 Delta | 21.480 | ns | **-** | 0.0118 | ns |
|  | SARS-CoV-2 Omicron | 36.800 | 0.0021 | 0.0118 | **-** | 0.0033 |
|  | SARS-CoV-2 614G | 21.130 | ns | ns | 0.0033 | **-** |
| **Nasal olfactory mucosa – IBA1 [% cells]** | | | |  |  |  |
|  | SARS-CoV-2 Gamma | 18.980 | **-** | ns | 0.0133 | ns |
|  | SARS-CoV-2 Delta | 18.760 |  | **-** | 0.0133 | ns |
|  | SARS-CoV-2 Omicron | 8.539 | 0.0133 | 0.0133 | **-** | 0.0185 |
|  | SARS-CoV-2 614G | 18.000 | ns | ns | 0.0185 | **-** |
| **Total nasal mucosa – MPO [% cells]** | | |  |  |  |  |
|  | SARS-CoV-2 Gamma | 2.716 | **-** | ns | 0.0027 | ns |
|  | SARS-CoV-2 Delta | 3.204 | ns | **-** | 0.0076 | ns |
|  | SARS-CoV-2 Omicron | 0.734 | 0.0027 | 0.0076 | **-** | ns |
|  | SARS-CoV-2 614G | 2.027 | ns | ns | ns | **-** |

**Supplementary Table 2 (continued): Result of statistical analysis of lesions and viral load in the nasal turbinates**: Group medians and exact p-values ≤0.05 obtained by two-tailed Mann-Whitney-U tests, followed by Benjamini-Hochberg-correction for multiple testing, are given.

| **Group** | **Median** | SARS-CoV-2 Gamma | SARS-CoV-2 Delta | SARS-CoV-2 Omicron | SARS-CoV-2 614G |
| --- | --- | --- | --- | --- | --- |

| **Nasal respiratory mucosa – MPO [% cells]** | | |  |  |  |  |
| --- | --- | --- | --- | --- | --- | --- |
|  | SARS-CoV-2 Gamma | 4.413 | **-** | ns | 0.0028 | ns |
|  | SARS-CoV-2 Delta | 4.715 | ns | **-** | ns | ns |
|  | SARS-CoV-2 Omicron | 1.760 | 0.0028 | ns | **-** | ns |
|  | SARS-CoV-2 614G | 2.665 | ns | ns | ns | **-** |

| **Nasal olfactory mucosa – MPO [% cells]** | | |  |  |  |  |
| --- | --- | --- | --- | --- | --- | --- |
|  | SARS-CoV-2 Gamma |  | **-** | ns | 0.0045 | ns |
|  | SARS-CoV-2 Delta |  | ns | **-** | 0.0197 | ns |
|  | SARS-CoV-2 Omicron |  | 0.0045 | 0.0197 | **-** | 0.0454 |
|  | SARS-CoV-2 614G |  | ns | ns | 0.0454 | **-** |

ns = not significant

**Supplementary Table 3: Result of statistical analysis of lesions and viral load in the trachea**: Group medians and exact p-values ≤0.05 obtained by two-tailed Mann-Whitney-U tests, followed by Benjamini-Hochberg-correction for multiple testing are given.

|  | **Group** | **Median** | SARS-CoV-2 Gamma | SARS-CoV-2 Delta | SARS-CoV-2 Omicron | SARS-CoV-2 614G |
| --- | --- | --- | --- | --- | --- | --- |
| **Trachea lesions** | |  |  |  |  |  |
|  | SARS-CoV-2 Gamma | 5 | **-** | ns | ns | ns |
|  | SARS-CoV-2 Delta | 4 | ns | **-** | ns | ns |
|  | SARS-CoV-2 Omicron | 4 | ns | ns | **-** | ns |
|  | SARS-CoV-2 614G | 4.5 | ns | ns | ns | **-** |
| **Trachea - SARS-CoV-2 NP [% cells]** | | | |  |  |  |
|  | SARS-CoV-2 Gamma | 0 | **-** | ns | 0.0079 | ns |
|  | SARS-CoV-2 Delta | 0 | ns | **-** | 0.0079 | ns |
|  | SARS-CoV-2 Omicron | 0.4881 | 0.0079 | 0.0079 | **-** | 0.0022 |
|  | SARS-CoV-2 614G | 0 | ns | ns | 0.0022 | **-** |

| **Trachea – IBA1 [% cells]** | | | |  |  |  |
| --- | --- | --- | --- | --- | --- | --- |
|  | SARS-CoV-2 Gamma | 20.320 | **-** | ns | ns | ns |
|  | SARS-CoV-2 Delta | 38.290 | ns | **-** | ns | ns |
|  | SARS-CoV-2 Omicron | 26.250 | ns | ns | **-** | ns |
|  | SARS-CoV-2 614G | 37.050 | ns | ns | ns | **-** |
| **Trachea – MPO [% cells]** | | | |  |  |  |
|  | SARS-CoV-2 Gamma | 0.335 | **-** | ns | ns | ns |
|  | SARS-CoV-2 Delta | 1.047 | ns | **-** | ns | ns |
|  | SARS-CoV-2 Omicron | 0.184 | ns | ns | **-** | ns |
|  | SARS-CoV-2 614G | 0.550 | ns | ns | ns | **-** |

ns = not significant

**Supplementary Table 4: Result of statistical analysis of lesions and viral load in the lung**: Group medians and exact p-values ≤0.05 obtained by two-tailed Mann-Whitney-U tests, followed by Benjamini-Hochberg-correction for multiple testing are given.

|  | **Group** | **Median** | SARS-CoV-2 Gamma | SARS-CoV-2 Delta | SARS-CoV-2 Omicron | SARS-CoV-2 614G |
| --- | --- | --- | --- | --- | --- | --- |
| **Total lung lesions** | |  |  |  |  |  |
|  | SARS-CoV-2 Gamma | 22 | **-** | ns | 0.0008 | ns |
|  | SARS-CoV-2 Delta | 21 |  | **-** | 0.0024 | ns |
|  | SARS-CoV-2 Omicron | 7.5 | 0.0008 | 0.0024 | **-** | 0.0230 |
|  | SARS-CoV-2 614G | 17 | ns | ns | 0.0230 | **-** |
| **Lung conductive airways lesions** | |  |  |  |  |  |
|  | SARS-CoV-2 Gamma | 8 | **-** | ns | 0.0009 | ns |
|  | SARS-CoV-2 Delta | 6 | ns | **-** | 0.0098 | ns |
|  | SARS-CoV-2 Omicron | 2.5 | 0.0009 | 0.0098 | **-** | 0.0228 |
|  | SARS-CoV-2 614G | 6 | ns | ns | 0.0228 | **-** |
| **Lung alveolar lesions** | |  |  |  |  |  |
|  | SARS-CoV-2 Gamma | 5 | **-** | ns | 0.0119 | ns |
|  | SARS-CoV-2 Delta | 8 | ns | **-** | 0.0025 | ns |
|  | SARS-CoV-2 Omicron | 1 | 0.0119 | 0.0025 | **-** | 0.0137 |
|  | SARS-CoV-2 614G | 5 | ns | ns | 0.0137 | **-** |
| **Lung vascular lesions** | |  |  |  |  |  |
|  | SARS-CoV-2 Gamma | 8 | **-** | ns | 0.0002 | ns |
|  | SARS-CoV-2 Delta | 6 |  | **-** | 0.0350 | ns |
|  | SARS-CoV-2 Omicron | 4 | 0.0002 | 0.0350 | **-** | 0.0093 |
|  | SARS-CoV-2 614G | 6.5 | ns | ns | 0.0093 | **-** |
| **Conductive airways - SARS-CoV-2 NP [% cells]** | | |  |  |  |  |
|  | SARS-CoV-2 Gamma | 1.008 | **-** | ns | 0.0362 | ns |
|  | SARS-CoV-2 Delta | 0.2386 | ns | **-** | ns | 0.0104 |
|  | SARS-CoV-2 Omicron | 0.2096 | 0.0362 | ns | **-** | 0.0022 |
|  | SARS-CoV-2 614G | 2.908 | ns | 0.0104 | 0.0022 | **-** |
|  | |  |  |  |  |  |

**Supplementary Table 4 (continued): Result of statistical analysis of lesions and viral load in the lung**: Group medians and exact p-values ≤0.05 obtained by two-tailed Mann-Whitney-U tests, followed by Benjamini-Hochberg-correction for multiple testing are given.

| **Group** | | **Median** | SARS-CoV-2 Gamma | SARS-CoV-2 Delta | SARS-CoV-2 Omicron | SARS-CoV-2 614G |
| --- | --- | --- | --- | --- | --- | --- |
| **Parenchyma - SARS-CoV-2 NP [% cells]** | |  |  |  |  |  |
|  | SARS-CoV-2 Gamma | 3.611 | **-** | ns | 0.0184 | ns |
|  | SARS-CoV-2 Delta | 4.282 | ns | **-** | 0.0275 | ns |
|  | SARS-CoV-2 Omicron | 0.6318 | 0.0184 | 0.0275 | **-** | ns |
|  | SARS-CoV-2 614G | 1.023 | ns | ns | ns | **-** |
| **Lung - TCID50 [TCID50/ml]** | |  |  |  |  |  |
|  | SARS-CoV-2 Gamma | 1780000 | **-** | 0.0297 | 0.001 | ns |
|  | SARS-CoV-2 Delta | 6810 | 0.0297 | **-** | ns | 0.0261 |
|  | SARS-CoV-2 Omicron | 2470 | 0.001 | ns | **-** | 0.0006 |
|  | SARS-CoV-2 614G | 1390000 | ns | 0.0261 | 0.0006 | **-** |
| **Lung – SARS-CoV-2 RNA [Ct]** | |  |  |  |  |  |
|  | SARS-CoV-2 Gamma | 14.79 | **-** | 0.0485 | 0.0002 | 0.0131 |
|  | SARS-CoV-2 Delta | 18.30 | 0.0485 | **-** | ns | ns |
|  | SARS-CoV-2 Omicron | 20.82 | 0.0002 | ns | **-** | ns |
|  | SARS-CoV-2 614G | 18.48 | 0.0131 | ns | ns | **-** |
| **Total lung - IBA1 [% cells]** | |  |  |  |  |  |
|  | SARS-CoV-2 Gamma | 35.430 | **-** | ns | 0.0039 | ns |
|  | SARS-CoV-2 Delta | 32.300 | ns | **-** | 0.0202 | ns |
|  | SARS-CoV-2 Omicron | 20.000 | 0.0039 | 0.0202 | **-** | ns |
|  | SARS-CoV-2 614G | 25.150 | ns | ns | ns | **-** |
| **Lung conductive airways –**  **IBA1 [% cells]** | |  |  |  |  |  |
|  | SARS-CoV-2 Gamma | 56.680 | **-** | ns | 0.0005 | ns |
|  | SARS-CoV-2 Delta | 44.390 | ns | **-** | ns | ns |
|  | SARS-CoV-2 Omicron | 26.350 | 0.0005 | ns | **-** | 0.0022 |
|  | SARS-CoV-2 614G | 56.360 | ns | ns | 0.0022 | **-** |

**Supplementary Table 4 (continued): Result of statistical analysis of lesions and viral load in the lung**: Group medians and exact p-values ≤0.05 obtained by two-tailed Mann-Whitney-U tests, followed by Benjamini-Hochberg-correction for multiple testing are given.

| **Group** | | **Median** | SARS-CoV-2 Gamma | SARS-CoV-2 Delta | SARS-CoV-2 Omicron | SARS-CoV-2 614G |
| --- | --- | --- | --- | --- | --- | --- |
| **Lung alveolar - IBA1 [% cells]** | |  |  |  |  |  |
|  | SARS-CoV-2 Gamma | 30.320 | **-** | ns | ns | ns |
|  | SARS-CoV-2 Delta | 30.000 | ns | **-** | ns | ns |
|  | SARS-CoV-2 Omicron | 19.70 | ns | ns | **-** | ns |
|  | SARS-CoV-2 614G | 21.510 | ns | ns | ns | **-** |
| **Lung vascular - IBA1 [% cells]** | |  |  |  |  |  |
|  | SARS-CoV-2 Gamma | 53.910 | **-** | ns | 0.0003 | ns |
|  | SARS-CoV-2 Delta | 44.800 | ns | **-** | 0.0026 | ns |
|  | SARS-CoV-2 Omicron | 17.760 | 0.0003 | 0.0026 | **-** | ns |
|  | SARS-CoV-2 614G | 34.920 | ns | ns | ns | **-** |
| **Total lung - MPO [% cells]** | |  |  |  |  |  |
|  | SARS-CoV-2 Gamma | 5.896 | **-** | ns | 0.0130 | 0.0036 |
|  | SARS-CoV-2 Delta | 5.268 | ns | **-** | 0.0334 | 0.0107 |
|  | SARS-CoV-2 Omicron | 3.466 | 0.0130 | 0.0334 | **-** | ns |
|  | SARS-CoV-2 614G | 2.752 | 0.0036 | 0.0107 | ns | **-** |
| **Lung conductive airways – MPO [% cells]** | | |  |  |  |  |
|  | SARS-CoV-2 Gamma | 7.356 | **-** | ns | 0.0226 | ns |
|  | SARS-CoV-2 Delta | 7.474 | ns | **-** | 0.0294 | ns |
|  | SARS-CoV-2 Omicron | 2.553 | 0.0226 | 0.0294 | **-** | ns |
|  | SARS-CoV-2 614G | 3.752 | ns | ns | ns | **-** |
| **Lung alveolar - MPO [% cells]** | |  |  |  |  |  |
|  | SARS-CoV-2 Gamma | 5.676 | **-** | ns | 0.0088 | 0.0010 |
|  | SARS-CoV-2 Delta | 4.736 | ns | **-** | ns | 0.0258 |
|  | SARS-CoV-2 Omicron | 3.459 | 0.0088 | ns | **-** | ns |
|  | SARS-CoV-2 614G | 2.651 | 0.0010 | 0.0258 | ns | **-** |

**Supplementary Table 4 (continued): Result of statistical analysis of lesions and viral load in the lung**: Group medians and exact p-values ≤0.05 obtained by two-tailed Mann-Whitney-U tests, followed by Benjamini-Hochberg-correction for multiple testing are given.

| **Group** | | **Median** | SARS-CoV-2 Gamma | SARS-CoV-2 Delta | SARS-CoV-2 Omicron | SARS-CoV-2 614G |
| --- | --- | --- | --- | --- | --- | --- |
| **Lung vascular - MPO [% cells]** | |  |  |  |  |  |
|  | SARS-CoV-2 Gamma | 6.022 | **-** | ns | ns | 0.0062 |
|  | SARS-CoV-2 Delta | 6.631 | ns | **-** | ns | 0.0097 |
|  | SARS-CoV-2 Omicron | 3.806 | ns | ns | **-** | ns |
|  | SARS-CoV-2 614G | 3.091 | 0.0062 | 0.0097 | ns | **-** |

**Conductive airways:** included bronchi, bronchioles and terminal bronchioles; ns = not significant

**Supplementary Table 5:** Semi-quantitative scoring system for evaluation of histopathological lesions in nose, trachea and lung of SARS-CoV-2 infected Syrian golden hamsters

| **Score** | **Grading** | **Description** |
| --- | --- | --- |
| 1. **Nose lesions** | | |
| - 1. **Nasal respiratory mucosa** | | |
| - - 1. **Inflammation of nasal respiratory mucosa:** | | |
| 0 | No - minimal | No inflammation to occasional foci with few inflammatory cells, less than 1% tissue |
| 1 | Mild | 2-25% of tissue affected |
| 2 | Moderate | 26-50% of tissue affected |
| 3 | Marked | 51-75% of tissue affected |
| 4 | Subtotal | > 75 % of tissue affected |
| - - 1. **Necrosis of nasal respiratory mucosa:** | | |
| 0 | No- minimal | no necrosis to occasional foci of few necrotic cells, less than 1% of tissue affected |
| 1 | Mild | 2-25% of tissue affected |
| 2 | Moderate | 26-50% of tissue affected |
| 3 | Marked | 51-75% of tissue affected |
| 4 | Subtotal | > 75 % of tissue affected |
| - - 1. **Epithelial hyper- and/or metaplasia of nasal respiratory mucosa** | | |
| 0 | No - minimal | No hyper-/metaplasia to occasional foci, less than 1% tissue |
| 1 | Mild | 2-25% of tissue affected |
| 2 | Moderate | 26-50% of tissue affected |
| 3 | Marked | 51-75% of tissue affected |
| 4 | Subtotal | > 75 % of tissue affected |
| - 1. **Nasal olfactory mucosa** | | |
| - - 1. **Inflammation of nasal olfactory mucosa** | | |
| 0 | No- minimal | No inflammation to occasional foci with few inflammatory cells, less than 1% of tissue affected |
| 1 | Mild | 2-25% of tissue affected |
| 2 | Moderate | 26-50% of tissue affected |
| 3 | Marked | 51-75% of tissue affected |
| 4 | Subtotal | > 75 % of tissue affected |
| - - 1. **Necrosis olfactory mucosa** | | |
| 0 | No - minimal | no necrosis to occasional foci of few necrotic cells, less than 1% of tissue affected |
| 1 | Mild | 2-25% of tissue affected |
| 2 | Moderate | 26-50% of tissue affected |
| 3 | Marked | 51-75% of tissue affected |
| 4 | Subtotal | > 75 % of tissue affected |
| - 1. **Intraluminal exudate** | | |
| 0 | No | no intraluminal exudate to exudat in less then 1 % of the lunmina |
| 1 | Mild | 2-25% of lumina affected |
| 2 | Moderate | 26-50% of lumina affected |
| 3 | Marked | 51-75% of lumina affected |
| 4 | Subtotal | > 75 % of lumina affected |

**Supplementary Table 5 (continued):** Semi-quantitative scoring system for evaluation of histopathological lesions in nose, trachea and lung of SARS-CoV-2 infected Syrian golden hamsters

| **Score** | **Grading** | **Description** |
| --- | --- | --- |
| - 1. **Vasculopathy (mural infiltrates, endothelial hypertrophy)** | | |
| 0 | No | no vasculopathy to mural infiltrates and/or endothelial hypertrophy in less then 1 % of the vessel |
| 1 | Mild | 2-25% of vessels affected |
| 2 | Moderate | 26-50% of vessels affected |
| 3 | Marked | 51-75% of vessels affected |
| 4 | Subtotal | > 75 % of vessels affected |

| 1. **Trachea lesion** | | |
| --- | --- | --- |
| - 1. **Extent trachea inflammation** | | |
| 0 | No | No inflammation |
| 1 | Mild | 2-25 % of tissue affected |
| 2 | Moderate | 26-50 % of tissue affected |
| 3 | Marked | 51-75 % of tissue affected |
| 4 | Subtotal | > 75 % of tissue affected |
| - 1. **Severity of tracheal inflammation (scored in area of maximal severity)** | | |
| 0 | No | No inflammation |
| 1 | Mild | Mild, mononuclear and granulocytic tracheitis (with exocytosis of inflammatory cells into epithelium, occasional single cell necrosis and mild, subepithelial infiltrates) |
| 2 | Moderate | Moderate, mononuclear and granulocytic to necrotizing tracheitis (with exocytosis of inflammatory cells into epithelium, frequent single cell necrosis and intraluminal debris and moderate, subepithelial infiltrates) |
| 3 | Marked | Marked, mononuclear and granulocytic and necrotizing tracheitis with widespread exocytosis of inflammatory cells into epithelium, frequent epithelial necrosis and intraluminal debris and severe, subepithelial infiltrates) |
| - 1. **Regeneration** | | |
| 0 | No - minimal | No lesions to single foci of mild hyperplasia, characterized by piling up of cells and increased number of mitoses, affecting max. 1% of tissue |
| 1 | Mild | Mild hyperplasia as above, 2-25 % of tissue affected |
| 2 | Moderate | Moderate hyperplasia as above, 26-50 % of tissue affected |
| 3 | Marked | Marked hyperplasia as above, 51-75 % of tissue affected |
| 4 | Subtotal | hyperplasia as above, >75 % of tissue affected |

**Supplementary Table 5 (continued):** Semi-quantitative scoring system for evaluation of histopathological lesions in nose, trachea and lung of SARS-CoV-2 infected Syrian golden hamsters

| **Score** | **Grading** | **Description** |
| --- | --- | --- |

| - 1. **Epithelial degeneration/necrosis** | | |
| --- | --- | --- |
| 0 | No- minimal | No lesions to single of mild degeneration/necrosis, characterized by vacuolization/cell shrinkage/ nuclear kariolysis, affecting max. 1% of tissue |
| 1 | Mild | Mild degeneration/necrosis as above, 2-25 % of tissue affected |
| 2 | Moderate | Moderate deg/nec as above, 26-50 % of tissue affected |
| 3 | Marked | Marked deg/nec as above, 51-75 % of tissue affected |
| 4 | Subtotal | Deg/nec as above, >75 % of tissue affected |
| - 1. **Intraluminal cell debris** | | |
| 0 | No | No cell debris |
| 1 | Mild | 2-25 % of tissue affected |
| 2 | Moderate | 26-50 % of tissue affected |
| 3 | Marked | 51-75 % of tissue affected |
| 4 | Subtotal | > 75 % of tissue affected |
| 1. **Lung** | | |
| - 1. **Alveolar lesions** | | |
| - - 1. **Extent alveolar inflammation** | | |
| 0 | No | No inflammation |
| 1 | Mild | 2-25 % of tissue affected |
| 2 | Moderate | 26-50 % of tissue affected |
| 3 | Marked | 51-75 % of tissue affected |
| 4 | Subtotal | > 75 % of tissue affected |
| - - 1. **Severity alveolar inflammation (scored in area of maximal severity)** | | |
| 0 | No | No inflammatory infiltrates |
| 1 | Minimal | Few inflammatory cells in alveolar septae or lumina, alveolar architecture maintained |
| 2 | Mild | Mild septal and luminal infiltrates (2-3 cells thick), alveolar architecture maintained |
| 3 | Moderate | Moderate septal and luminal infiltrates, occasionally obscuring alveolar architecture |
| 4 | Marked | Marked septal and luminal infiltrates with areas with completely obscured alveolar architecture |

**Supplementary Table 5 (continued):** Semi-quantitative scoring system for evaluation of histopathological lesions in nose, trachea and lung of SARS-CoV-2 infected Syrian golden hamsters

| **Score** | **Grading** | **Description** |
| --- | --- | --- |

| - - 1. **Alveolar necrosis/desquamation/loss** | | |
| --- | --- | --- |
| 0 | No | No lesions |
| 1 | Minimal | Minimal alveolar necrosis |
| 2 | Mild | Mild, multifocal alveolar necrosis |
| 3 | Moderate | Moderate, multifocal to coalescing alveolar necrosis |
| 4 | Marked | Marked, multifocal to coalescing alveolar necrosis |
| - - 1. **Intraalveolar fibrin/hyaline membranes** | | |
| 0 | No | No lesions |
| 1 | Minimal | Minimal intraalveolar fibrin |
| 2 | Mild | Mild, multifocal intraalveolar fibrin |
| 3 | Moderate | Moderate, multifocal intraalveolar fibrin |
| 4 | Marked | Marked, multifocal intraalveolar fibrin |
| - - 1. **Atypical/large or multinucleated cells lining alveoli** | | |
| 0 | No | No multinucleated cells |
| 1 | Minimal | Few multinucleated cells |
| 2 | Mild | Mild multinucleated infiltrates |
| 3 | Moderate | Moderate, multifocal multinucleated infiltrates |
| 4 | Marked | Marked, multifocal multinucleated infiltrates |
| - - 1. **Alveolar edema** | | |
| 0 | No | No lesions |
| 1 | Mild | 2-25 % of alveoli affected |
| 2 | Moderate | 26-50 % of alveoli affected |
| 3 | Marked | 51-75 % of alveoli affected |
| 4 | Subtotal | > 75 % of alveoli affected |
| - - 1. **Alveolar hemorrhage** | | |
| 0 | No | No lesions |
| 1 | Mild | 2-25 % of alveoli affected |
| 2 | Moderate | 26-50 % of alveoli affected |
| 3 | Marked | 51-75 % of alveoli affected |
| 4 | Subtotal | > 75 % of alveoli affected |

**Supplementary Table 5 (continued):** Semi-quantitative scoring system for evaluation of histopathological lesions in nose, trachea and lung of SARS-CoV-2 infected Syrian golden hamsters

| **Score** | **Grading** | **Description** |
| --- | --- | --- |

| - - 1. **Extent alveolar regeneration** | | |
| --- | --- | --- |
| 0 | No | No alveolar regeneration |
| 1 | Mild | Mild, multifocal epithelial hyperplasia with atypical/large/multinucleated cells/affecting 2-25 % of tissue |
| 2 | Moderate | Moderate, multifocal to coalescing epithelial hyperplasia with atypical/large/multinucleated cells/affecting 26-50 % of tissue |
| 3 | Marked | Marked, multifocal to coalescing epithelial hyperplasia with atypical/large/multinucleated cells/affecting 51-75 % of tissue |
| 4 | Subtotal | Subtotal epithelial hyperplasia with atypical/large/multinucleated cells/affecting >75 % of tissue |
| - 1. **Conductive airway lesions** | | |
| - - 1. **Extent of conductive airway inflammation** | | |
| 0 | No | No airway inflammation |
| 1 | Mild | 2-25 % of tissue affected |
| 2 | Moderate | 26-50 % of tissue affected |
| 3 | Marked | 51-75 % of tissue affected |
| 4 | Subtotal | > 75 % of tissue affected |
| - - 1. **Severity of conductive airway inflammation (scored in area of maximal severity)** | | |
| 0 | No | No inflammation |
| 1 | Minimal | Rare peribronchial/peribronchiolar, mild, mononuclear infiltrates |
| 2 | Mild | Mild, mononuclear and granulocytic bronchitis/bronchiolitis (with exocytosis of inflammatory cells into epithelium, occasional single cell necrosis and mild, peribronchial/peribronchiolar infiltrates) |
| 3 | Moderate | Moderate, mononuclear and granulocytic to necrotizing bronchitis/bronchiolitis (with exocytosis of inflammatory cells into epithelium, frequent single cell necrosis and intraluminal debris and moderate, peribronchial/peribronchiolar infiltrates) |
| 4 | Marked | Marked, mononuclear and granulocytic and necrotizing bronchitis/bronchiolitis with widespread exocytosis of inflammatory cells into epithelium, frequent epithelial necrosis and intraluminal debris and severe, peribronchial/peribronchiolar infiltrates) |

**Supplementary Table 5 (continued):** Semi-quantitative scoring system for evaluation of histopathological lesions in nose, trachea and lung of SARS-CoV-2 infected Syrian golden hamsters

| **Score** | **Grading** | **Description** |
| --- | --- | --- |

| Regeneration | | | |
| --- | --- | --- | --- |
| 0 | No | No regeneration | |
| 1 | Mild | Mild hyperplasia as above, 2-25 % of tissue affected | |
| 2 | Moderate | Moderate hyperplasia as above, 26-50 % of tissue affected | |
| 3 | Marked | Marked hyperplasia as above, 51-75 % of tissue affected | |
| 4 | Subtotal | Subtotal hyperplasia as above, >75% of tissue affected | |
| Vascular lesions | | | |
| Extent vasculopathy/vasculitis (characterized by endothelialitis and mural infiltrates with disruption of vessel walls) | | | |
| 0 | No | No lesions | |
| 1 | Mild | 2-25 % of vessels affected | |
| 2 | Moderate | 26-50 % of vessels affected | |
| 3 | Marked | 51-75 % of vessels affected | |
| 4 | Subtotal | > 75 % of vessels affected | |
| Severity vasculitis/perivasculitis (scored in area of maximal severity) | | |  |
| 0 | No | No inflammation |  |
| 1 | Minimal | Single vessels with endothelial hypertrophy and few perivascular cells (no continuous cuff) |  |
| 2 | Mild | 1-2 cell layers of perivascular cuffs |  |
| 3 | Moderate | 3-5 cell layers of perivascular cuffs |  |
| 4 | Marked | > 5 cell layers of perivascular cuffs |  |
| Perivascular edema | | |  |
| 0 | No - minimal | No lesions to single vessels with mild edema |  |
| 1 | Mild | 2-25 % of vessels affected |  |
| 2 | Moderate | 26-50 % of vessels affected |  |
| 3 | Marked | 51-75 % of vessels affected |  |
| 4 | Subtotal | > 75 % of vessels affected |  |
| Perivascular hemorrhage | | |  |
| 0 | No - minimal | No lesions to single vessels with mild hemorrhage |  |
| 1 | Mild | 2-25 % of vessels affected |  |
| 2 | Moderate | 26-50 % of vessels affected |  |
| 3 | Marked | 51-75 % of vessels affected |  |
| 4 | Subtotal | > 75 % of vessels affected |  |
