# Supplementary material for: SARS-CoV-2 Omicron variant causes mild pathology in the upper and lower respiratory tract of hamsters
Source: Nat Commun. 2022 Jun 20;13:3519. doi: 10.1038/s41467-022-31200-y (PMC9207884; doi:10.1038/s41467-022-31200-y)
Supplement: Supplementary file 3 — Reporting Summary [file 41467_2022_31200_MOESM3_ESM.pdf]

nature portfolio

Corresponding author(s): Jacob J. Bukoski Ph.D.

Last updated by author(s): Jan 7, 2022

Reporting Summary

Nature Portfolio wishes to improve the reproducibility of the work that we publish. This form provides structure for consistency and transparency in reporting. For further information on Nature Portfolio policies, see our Editorial Policies and the Editorial Policy Checklist.

Statistics

For all statistical analyses, confirm that the following items are present in the figure legend, table legend, main text, or Methods section.

n/a	Confirmed	
☐	☒	The exact sample size (*n*) for each experimental group/condition, given as a discrete number and unit of measurement
☒	☐	A statement on whether measurements were taken from distinct samples or whether the same sample was measured repeatedly
☐	☒	The statistical test(s) used AND whether they are one- or two-sided<br>*Only common tests should be described solely by name; describe more complex techniques in the Methods section.*
☐	☒	A description of all covariates tested
☐	☒	A description of any assumptions or corrections, such as tests of normality and adjustment for multiple comparisons
☐	☒	A full description of the statistical parameters including central tendency (e.g. means) or other basic estimates (e.g. regression coefficient) AND variation (e.g. standard deviation) or associated estimates of uncertainty (e.g. confidence intervals)
☐	☒	For null hypothesis testing, the test statistic (e.g. *F*, *t*, *r*) with confidence intervals, effect sizes, degrees of freedom and *P* value noted<br>*Give P values as exact values whenever suitable.*
☒	☐	For Bayesian analysis, information on the choice of priors and Markov chain Monte Carlo settings
☐	☒	For hierarchical and complex designs, identification of the appropriate level for tests and full reporting of outcomes
☐	☒	Estimates of effect sizes (e.g. Cohen's *d*, Pearson's *r*), indicating how they were calculated

Our web collection on statistics for biologists contains articles on many of the points above.

Software and code

Policy information about availability of computer code

Data collection	No code was used in the data collection component of this research.
Data analysis	All analyses were performed in Program R (v 4.0.4). All scripts were written by the authors of this study. All code to reproduce the analyses and statistics will be made available through a GitHub repository associated with this study. We have provided the versions of all R packages in the R scripts themselves. Key R packages for our analysis included lmerTest (v 3.1.3), nlme (v 3.1.153), and saemix (v 2.4).

For manuscripts utilizing custom algorithms or software that are central to the research but not yet described in published literature, software must be made available to editors and reviewers. We strongly encourage code deposition in a community repository (e.g. GitHub). See the Nature Portfolio guidelines for submitting code & software for further information.

Data

Policy information about availability of data

All manuscripts must include a data availability statement. This statement should provide the following information, where applicable:

- Accession codes, unique identifiers, or web links for publicly available datasets
- A description of any restrictions on data availability
- For clinical datasets or third party data, please ensure that the statement adheres to our policy

The monoculture plantations aboveground carbon stock data compiled in this study are published on Zenodo (https://doi.org/10.5281/zenodo.6555216). A publicly facing version of the Spatial Database of Planted Trees is available through the World Resources Institute (WRI), whereas the version used here may be requested through Global Forest Watch at WRI. The Global Wood Density Database is available through DataDryad (https://doi.org/10.5061/dryad.234). The plant trait data were accessed through the TRY Plant Trait Database (https://www.try-db.org/TryWeb/Home.php). The spatial information on biomes of the globe were obtained through the RESOLVE Ecoregions 2017 database (https://ecoregions.appspot.com/). The Food and Agriculture Organization of the United Nation's Global Ecological

Zones are available through the FAO's data center (https://data.review.fao.org/map/catalog/srv/search?keyword=FRA). J.J.B welcomes discussions around potential collaborations in using and expanding the data published here.

Field-specific reporting

Please select the one below that is the best fit for your research. If you are not sure, read the appropriate sections before making your selection.

☐ Life sciences ☐ Behavioural & social sciences ☒ Ecological, evolutionary & environmental sciences

For a reference copy of the document with all sections, see nature.com/documents/nr-reporting-summary-flat.pdf

Ecological, evolutionary & environmental sciences study design

All studies must disclose on these points even when the disclosure is negative.

Study description	We systematically reviewed the literature to identify studies reporting aboveground carbon stocks in monoculture forest plantations. Using these data, we then assessed a suite of potential drivers of variation in carbon stocks and rates of carbon stocking as well as parameterized nonlinear growth functions (Chapman-Richards curves) using the data.
Research sample	Our research sample is a database of aboveground carbon stocks, stand age, location, and a suite of covariates for monoculture plantations across the globe. The sample is believed to be representative of plantations globally, although subsets of our database may be non-representative for specific taxa. Our database includes observations for the dominant genera of trees planted in monoculture plantations including (but not limited to) Pinus, Eucalyptus, Cunninghamia, Populus, Acacia, Larix, Picea, Tectona, Castanea, and Quercus. We provide detailed discussion of how representative of global monoculture plantations our database is, as well as recommend additional work to improve its representativeness.
Sampling strategy	We did not predetermine a sample size, but exhaustively reviewed the scientific literature published prior to April 19, 2017. Our database holds ~ 5,000 observations of aboveground carbon stocks in monoculture plantations.
Data collection	We (J. Bukoski, C. Melikov, J. Liu, S. Ban, S. C. Cook-Patton) systematically reviewed studies returned by our literature search to identify those reporting i) empirical measurements of biomass or carbon in the aboveground pool, ii) age of the plantation at the time of field measurements, and iii) a latitude or longitude pair or sufficient geographic detail from which geographic coordinates could be obtained. We reviewed titles and abstracts first to identify those studies reporting carbon stocks in plantations. From this subset, we then read the papers to identify whether the studies met our criteria for inclusion. We then manually extracted data on location, carbon stocks, age, and a suite of management covariates that we included in our driver analysis. We compiled the extracted data in a Microsoft Excel Spreadsheet, which is published on Zenodo (https://doi.org/10.5281/zenodo.6555216). We used basic functions in Program R to clean the compiled dataset.
Timing and spatial scale	Our literature search was performed on April 19, 2017. Only studies that were published and indexed in Web of Science were included in our search results. The spatial scale of our study was global.
Data exclusions	Data exclusions were performed during the article screening process. That is, studies that did not meet our inclusion criteria (described in the Data Collection box) were excluded from future consideration.
Reproducibility	Upon downloading the publicly available dataset and associated code on GitHub, all analyses are reproducible.
Randomization	No randomization was used given that we conducted a systematic review of the literature. We included all observations in our analyses.
Blinding	We did not use blinding (e.g., withholding author names of the reviewed studies from the reviewers) as unconscious biases would not impact our recording of aboveground carbon stock measurements into an excel sheet. The expected influence of having all study information available to the reviewers is likely to have negligible impact on our results.

Did the study involve field work? ☐ Yes ☒ No

Reporting for specific materials, systems and methods

We require information from authors about some types of materials, experimental systems and methods used in many studies. Here, indicate whether each material, system or method listed is relevant to your study. If you are not sure if a list item applies to your research, read the appropriate section before selecting a response.

Materials & experimental systems

n/a	Involved in the study
☒	☐ Antibodies
☒	☐ Eukaryotic cell lines
☒	☐ Palaeontology and archaeology
☒	☐ Animals and other organisms
☒	☐ Human research participants
☒	☐ Clinical data
☒	☐ Dual use research of concern

Methods

n/a	Involved in the study
☒	☐ ChIP-seq
☒	☐ Flow cytometry
☒	☐ MRI-based neuroimaging
